# Potential Neurophysiological Mechanisms of 1Hz-TMS to the Right Prefrontal Cortex for Depression: An Exploratory TMS-EEG Study in Healthy Participants

**DOI:** 10.3390/jpm11020068

**Published:** 2021-01-24

**Authors:** Yoshihiro Noda

**Affiliations:** Department of Neuropsychiatry, Graduate School of Medicine, Keio University School of Medicine, 35 Shinanomachi, Shinjuku-ku, Tokyo 160-8582, Japan; yoshi-tms@keio.jp; Tel.: +81-3-3353-1211 (ext. 61857)

**Keywords:** dorsolateral prefrontal cortex (DLPFC), electroencephalography (EEG), neuromodulation, neurophysiology, transcranial magnetic stimulation (TMS), TMS-EEG, TMS-evoked potential (TEP), TMS-related coherence, TMS-related power

## Abstract

Background: The present study aimed to examine the acute neurophysiological effects of 1Hz transcranial magnetic stimulation (TMS) administered to the right dorsolateral prefrontal cortex (DLPFC) in healthy participants. Methods: TMS combined with simultaneous electroencephalography (EEG) recording was conducted for 21 healthy participants. For the right DLPFC, 1Hz-TMS (100 pulses/block × 17 sessions) was applied in the resting-state, while for the left DLPFC, 1Hz-TMS (100 pulses/block × 2 sessions) was administered during the verbal fluency tasks (VFTs). For TMS-EEG data, independent component analysis (ICA) was applied to extract TMS-evoked EEG potentials to calculate TMS-related power as well as TMS-related coherence from the F4 and F3 electrode sites during the resting-state and VFTs. Results: TMS-related power was significantly increased in alpha, beta, and gamma bands by 1Hz-TMS at the stimulation site during the resting-state, while TMS-related power was significantly increased in alpha and beta bands but not in the gamma band during the VFTs. On the other hand, TMS-related coherence in alpha and beta bands significantly increased but not in gamma band by 1Hz-TMS that was administered to the right DLPFC in resting-state, whereas there were no significant changes in coherence for all frequency bands by 1Hz-TMS that applied to the left DLPFC during the VFTs. Conclusions: Collectively, 1Hz-repetitive TMS (rTMS) to the right DLPFC may rapidly neuromodulate EEG activity, which might be associated with a therapeutic mechanism for depression.

**Highlights**

- Low-frequency 1Hz-TMS to the right DLPFC in healthy participants resulted in a significant increase in spectral power in alpha, beta, and gamma bands.
- The same 1Hz-TMS induced a significant increase in alpha and beta coherences between the right and left DLPFC in healthy participants.
- These findings suggest parts of the therapeutic mechanisms of 1Hz-rTMS to the right DLPFC which may exert in patients with depression.

## 1. Introduction

Repetitive transcranial magnetic stimulation (rTMS) to the dorsolateral prefrontal cortex (DLPFC) in major depression can improve clinical symptoms and cognitive function as shown in previous clinical studies [1,2,3]. However, the neurophysiological therapeutic mechanisms are not fully understood [4,5,6]. On the other hand, most previous studies that examined the neurophysiology of healthy subjects focused on the motor cortex and demonstrated changes in electroencephalography (EEG) power and EEG coherence in the motor cortex [7,8,9,10,11], but few studies have examined the EEG changes induced by rTMS administered to the prefrontal cortex [12], which also implies that the therapeutic mechanism induced by rTMS in depression has not been fully elucidated. 

Specifically, previous studies have shown that patients with depression have lower activity in the left prefrontal cortex and relatively higher activity in the right prefrontal cortex, suggesting that an imbalance between the activity of the left and right prefrontal cortex may be associated with the pathophysiology of depression [13,14,15]. Given the clinical effects of low-frequency rTMS of the right DLPFC on depression, it may have the effect of normalizing the imbalance of right and left prefrontal activity. Here, the present study aimed to investigate the prefrontal EEG activity changes induced by low-frequency rTMS over the right DLPFC as indexed by the functional connectivity between right and left DLPFC as well as the EEG power changes in the prefrontal cortex in healthy participants, using TMS-compatible EEG equipment [16,17,18,19,20,21]. 

Thereupon, it was speculated that the EEG aftereffects of low-frequency rTMS to the right DLPFC [22] may be detected as changes in EEG power in the prefrontal cortex and changes in EEG coherence between the left and right DLPFC [23]. The present study aimed to explore such neurophysiological therapeutic mechanisms, assuming that 1Hz-rTMS for the right DLPFC would cause EEG changes in healthy participants that would be partially similar to the effects on depressed patients [24,25].

## 2. Material and Methods

### 2.1. Participants

Twenty-one healthy young adults (11 males and 10 females; mean age ± S.D = 34 ± 6 years) participated in the study. Participants reported no neurological, psychiatric, or other excluding medical conditions. A trained psychiatrist administered a short structured diagnostic interview using the Mini-international Neuropsychiatric Interview (M.N.N.I.) [26,27] to confirm that they did not have a lifetime diagnosis of affective, anxiety or psychotic disorders based on the criteria of the Diagnostic and Statistical Manual of Mental Disorders, 4th edition (DSM-IV). The study was approved by the local ethics committee of Kanagawa Psychiatric Center and was conducted under the principles laid down in the Declaration of Helsinki. All participants provided written informed consent.

### 2.2. TMS and EEG System

TMS was conducted with a Magstim Rapid system (The Magstim Company Ltd., Spring Gardens, Whitland, UK). TMS was delivered through a figure-of-8-shaped coil (70 mm diameter; Magstim). TMS-compatible EEG system (NEURO PRAX, neuroConn Gmbh, Ilmenau, Germany) which can record the EEG data at a sampling rate of 4096Hz. The EEGs were continuously acquired from 19 recording electrode sites (i.e., Fp1, Fp2, F7, F3, Fz, F4, F8, T3, C3, Cz, C4, T4, T5, P3, Pz, P4, T6, O1, and O2) according to the International 10–20 System. An elastic cap with small Ag/AgCl pellet electrodes was used to avoid overheats due to TMS. The ground electrode was positioned at Fpz, and linked earlobes (A1 and A2) served as the active reference for all the electrodes. Skin–electrode impedance was maintained below 5 kΩ during the EEG recording.

### 2.3. TMS Experimental Procedure

First, each participant’s resting motor threshold (RMT) was measured. RMT was defined as the lowest stimulus intensity produced in the relaxed right abductor digiti minimi (ADM) muscle at least 5 motor-evoked potentials (MEPs) over 50 μV in 10 consecutive trials [28]. Next, TMS stimulation was applied to the F4 electrode site on the right DLPFC during the resting-state (17 blocks as described below) at various stimulus intensities, while TMS stimulation was administered to the F3 electrode site on the left DLPFC during the verbal fluency task (VFT) (two blocks as described below) at the RMT intensity in all participants. Specifically, the protocol for TMS in the resting-state consisted of a set of 100 s of 1Hz stimulation (i.e., 100 pulses per block) for F4 electrodes in 5% increments from 10% to 90% maximum stimulus output (MSO) (10%, 15%,..., 90%), for a total of 17 blocks of 1700 pulses to obtain the recruitment curve (i.e., input–output curve) of TMS-evoked potentials (TEPs). On the other hand, the TMS protocol during VFTs (VFT-1 and VFT-2) was performed as a set of 1Hz stimulation for 100 s (i.e., a total of 200 pulses in two blocks). During the TMS-EEG measurement experiment, the participants were instructed to remain in a resting-state as in the standard EEG experiment, not to move or talk, and to remain awake and close their eyes lightly.

### 2.4. TMS-EEG Experiment during the Verbal Fluency Task (VFT)

The VFT was conducted after the recruitment curve experiment to examine the synergy effect between VFT and TMS. The VFT-1 was the task that participants were instructed to vocalize *hiragana* in order such as “a, i, u, e, o, ka, ki, ku, ke, ko,….” during the 1Hz TMS for 100 s over the left DLPFC. The VFT-2 task was that participants were instructed to say as many common nouns as they could think of beginning with “a” (e.g., asa, aka, ao, aki, and so on), next with “i” (e.g., ike, isi, ika, and so on), then “u”, in *hiragana* order, during 1Hz-TMS for 100 s. In this study, the number of words were not counted, because these tasks were intended to apply a verbal cognitive load to the participants during TMS stimulation over the left DLPFC.

### 2.5. EEG Signal Processing

EEG data were exported from the EEG device as European data format (EDF) files and processed offline using the EEGLAB toolbox [29] running in MATLAB software (Mathworks, Natic, MA, USA). The EEG signal was processed in accordance with the published methodology [30,31]. All EEG data were epoched from −500 ms to 500 ms relative to the TMS pulse and further baseline correction was performed for the pre-stimulus interval −300 ms to −110 ms. Then, EEG data were visually double-checked to exclude bad channels and trials. Next, independent component analysis (ICA) was applied to minimize and remove the typical TMS–related decay artifacts and eye-related and muscle activity-related components [32,33,34,35]. Afterwards, a notch filter was applied to avoid the power-line noise of 50Hz. Further, the processed data were down-sampled to 500Hz and then bandpass filtered between 1 and 100Hz. The number of ICA components that were removed from the original 19 IC was no greater than 20% in each participant. Finally, the data were re-referenced to an average of both earlobes’ electrodes (A1 and A2). 

### 2.6. ICA Technique to Remove TMS-Related Artifacts

The ICA method was applied to remove TMS-related artifacts, which can separate statistically independent sources from a mixed signal. ICA is ideally suited to separate the electrical artifacts from physiological data in the EEG recordings and this technique has already been successfully applied to remove other non-neurophysiological EEG artifacts in previous studies [33,34,35,36]. Residual TMS artifacts such as eye-blinks and some muscle activity contractions were identified and removed with ICA as implemented in EEGLAB [29]. Two rounds of ICAs were conducted on the EEG data according to the previous study [34]. TMS-related artifact components were identified based on the following characteristics. First, the power spectrum of ICs that shows an extremely strong power, above 50 μV, presumably due to muscle activity contractions. Second, the power spectrum of ICs that shows peaks at over 100Hz (e.g., mechanical-like shaped peaks), potentially due to muscle activities as well, and do not usually have a peak at around 10Hz (the α-band peak). A third criterion concerned the component activity as follows. If the TMS-related artifact is present, it is limited to the first 15 ms (~30 ms at most) after the TMS pulse with the TMS-compatible EEG system [16,36,37]. Therefore, ideally, the ICs reflecting the TMS-related decay artifacts should peak within a few milliseconds of the start of TMS pulse [34].

### 2.7. Data Analysis

First, the amplitudes of N100 TEP for each intensity from 10% to 90% MSO were analyzed to obtain the recruitment curve. Then, the event-locked analysis was conducted by averaging 100 times for each intensity to obtain TEPs. EEG powers at the stimulation site (F4 site for the recruitment curve experiment and F3 site for the VFTs) were calculated before and after 1Hz-TMS during the resting-state. Here, since the time window after TMS stimulation was short (~500 ms), the event-related power was analyzed at an intensity close to the RMT (approximately 70% MSO intensity on average) of each patient from alpha (8–14Hz) to gamma (30–70Hz) bands considering a reliable margin. In other words, the spectral power of the delta and theta bands could not be calculated in the present analysis because of the narrow time window after TMS.

Next, the coherence analyses were conducted using the EEGLAB toolbox for both the baseline and after the TMS stimulation of each block. In the coherence analysis, the functional connectivity between the F4 and F3 electrode sites during the TMS was calculated to explore the neurophysiological and potential therapeutic mechanism for depression, since 1Hz-rTMS over the right DLPFC is often applied for this disorder as therapeutic neuromodulation. Specifically, the average coherence values in the alpha (8–14Hz), beta (15–30Hz), and gamma (30–50Hz) bands between the F4–F3 electrodes in the RMT of each subject were calculated.

### 2.8. Statistical Analysis

A one-way repeated measure analysis of variance (ANOVA) with time was performed using SPSS Statistics 19 (IBM, Armonk, NY, USA) for the changes in power and coherence before and after TMS according to each frequency band. Of note, Bonferroni correction was applied in this study and thus the significance level α was set to 0.0083 (=0.05/6: three frequency bands by two VFTs).

## 3. Results

### 3.1. Recruitment Curve of TEPs

**Figure 1:**
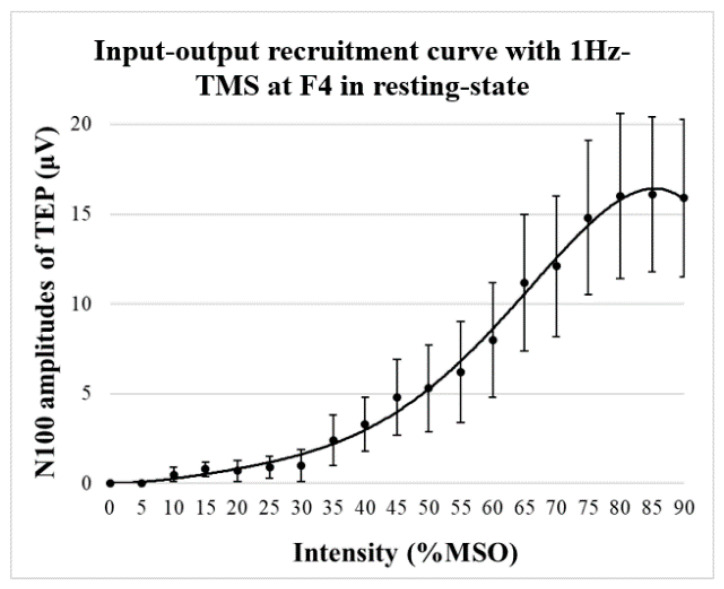
Recruitment curve of N100 component of TMS-evoked potentials (TEPs) at the stimulation site. The graph shows the recruitment curve at the F4 electrode site where 1Hz-TMS was administered. It shows that the input-output (I-O) curve increases nonlinearly with intensity and plateaus at an intensity of 80% maximum stimulus output (MSO).

The mean RMT in this study was 68 ± 11%. TMS-evoked potentials from the F4 electrode site during the resting-state were calculated and the recruitment (input–output) curve was created (Figure 1). The input–output (I–O) curve increased nonlinearly with intensity, reaching a plateau at approximately 80% MSO intensity in the present study of healthy participants. 

### 3.2. TMS-Related Power Changes at the F4 Electrode Site

#### 3.2.1. Results in Resting-State

**Figure 2:**
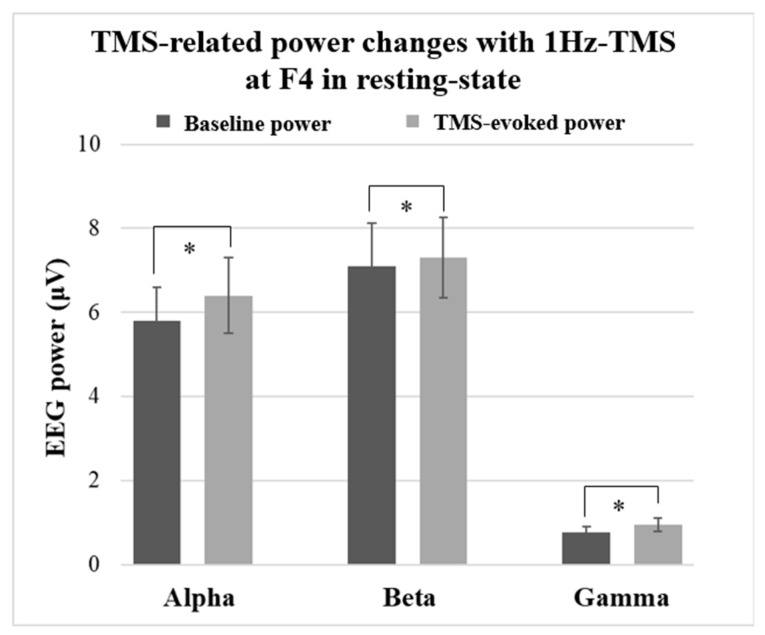
TMS-related power changes at F3 in the resting-state. The spectral power changes at the F4 electrode site after 100 pulses of 1Hz-TMS to the right dorsolateral prefrontal cortex (DLPFC) showed 10.2% increase in the alpha band (F_1,20_ = 132.4, *p* < 0.0001), 2.2% increase in the beta band (F_1,20_ = 1824.7, *p* < 0.0001), and 51% increase in the gamma band (F_1,20_ = 1028.5, *p* < 0.0001). *: significant findings (*p* < 0.0083).

One-way ANOVA with time before and after the TMS for each frequency band indicated the following results. The spectral power at the F4 site indicated 10.2% increase in alpha band (F_1,20_ = 132.4, *p* < 0.0001), 2.2% increase in beta band (F_1,20_ = 1824.7, *p* < 0.0001), and 51% increase in gamma band (F_1,20_ = 1028.5, *p* < 0.0001) after 100 times 1Hz-TMS to the right DLPFC (F4 electrode site). Figure 2 shows the results of EEG power changes at F4 electrode site induced by 1Hz-TMS (100 pulses/block).

#### 3.2.2. Results during VFTs

**Figure 3:**
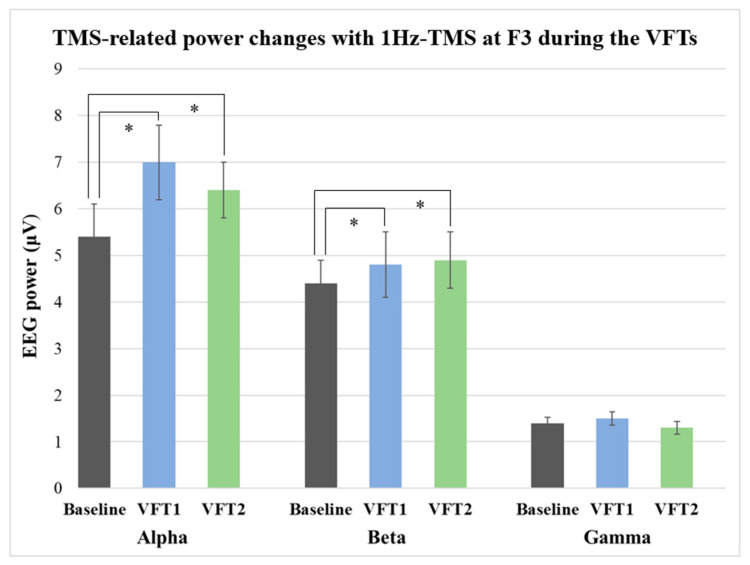
TMS-related power changes during the verbal fluency tasks (VFTs). One-way ANOVA with time for each band demonstrated significant power changes with 1Hz-TMS administered to the left DLPFC in alpha band during the VFT-1 (F_1,20_ = 53.96, *p* < 0.0001) as well as during the VFT-2 (F_1,20_ = 76.68, *p* < 0.0001). Furthermore, the ANOVA indicated significant power changes with 1Hz-TMS for the left DLPFC in beta band during the VFT-1 (F_1,20_ = 20.79, *p* = 0.0001) as well as during the VFT-2 (F_1,20_ = 13.00, *p* = 0.004). However, no significant changes were observed in gamma power during the VFT-1 or VFT-2. *: significant findings (*p* < 0.0083).

One-way ANOVA with time for each band demonstrated significant power changes in alpha band during the VFT-1 (F_1,20_ = 54.0, *p* < 0.0001) as well as VFT-2 (F_1,20_ = 76.7, *p* < 0.0001) by 1Hz-TMS (100 pulses/block) over the left DLPFC (F3 electrode site). Further, the ANOVA also indicated significant power changes by 1Hz-TMS (100 pulses/block) in beta band during the VFT-1 (F_1,20_ = 20.8, *p* = 0.0001) as well as VFT-2 (F_1,20_ = 13.0, *p* = 0.004). However, no significant changes were observed in gamma band during VFT-1 (F_1,20_ = 0.039, *p* = 0.847) or VFT-2 (F_1,20_ = 0.369, *p* = 0.555). Furthermore, no significant difference was found between TMS-related power in each frequency band during VFT-1 and VFT-2 administrations. Figure 3 shows the results of TMS-related power changes at F3 electrode site during the resting-state and VFTs.

### 3.3. TMS-Related Coherence Changes between F4 and F3 Electrode Sites

#### 3.3.1. Results in Resting-State

**Figure 4:**
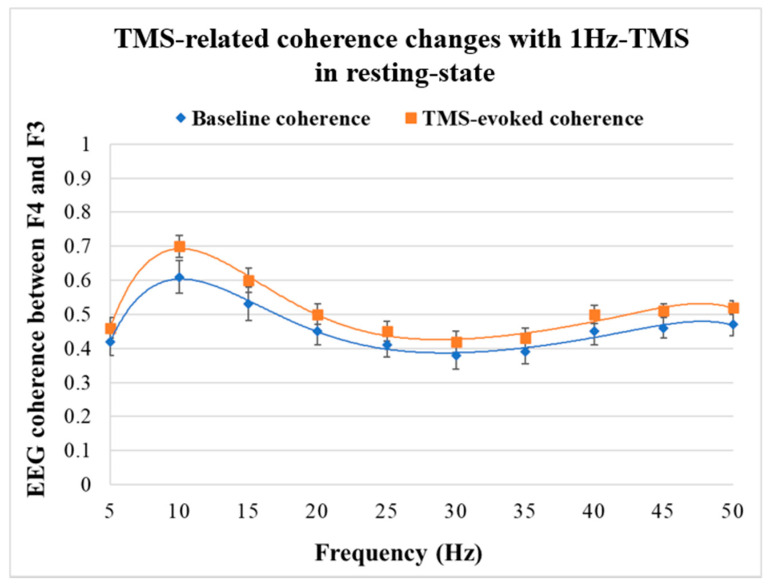
TMS-related coherence changes between F4 and F3 with 1Hz-TMS in resting-state.

Figure 4 shows the coherence-frequency curves and coherence changes induced by 1Hz-TMS applied to the right DLPFC (F4 electrode site). 

Figure 4 depicts the EEG coherence-frequency curves before and after 1Hz-TMS stimulation. In this experiment, TMS was applied to the right DLPFC (i.e., F4 electrode site) and the coherences between F4 and F3 electrodes was calculated.

**Figure 5:**
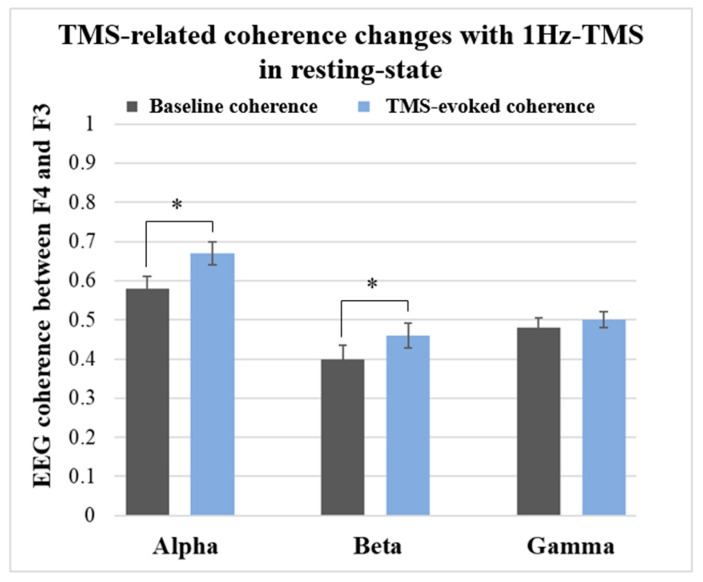
TMS-related coherence changes in the resting-state. The ANOVA showed significant changes of coherence in alpha band (F_1,20_ = 78.8, *p* < 0.0001) and beta band (F_1,20_ = 26.2, *p* < 0.0001) but not in gamma band (F_1,20_ = 11.6, *p* = 0.003) in resting-state. *: significant findings (*p* < 0.0083).

One-way ANOVA with time for each band revealed significant coherence changes in alpha (F_1,20_ = 78.8, *p* < 0.0001), and beta band (F_1,20_ = 26.2, *p* < 0.0001). However, the ANOVA did not show a significant coherence change in gamma band (F_1,20_ = 11.6, *p* = 0.003). The descriptive data showed a significant increase in alpha and beta coherences and an increasing trend in gamma coherence. Figure 5 shows the results of TMS-related coherence changes between F4 and F3 electrode sites with 1Hz-TMS that applied to the right DLPFC (i.e., F4 electrode site) in the resting-state.

#### 3.3.2. Results during VFTs

**Figure 6:**
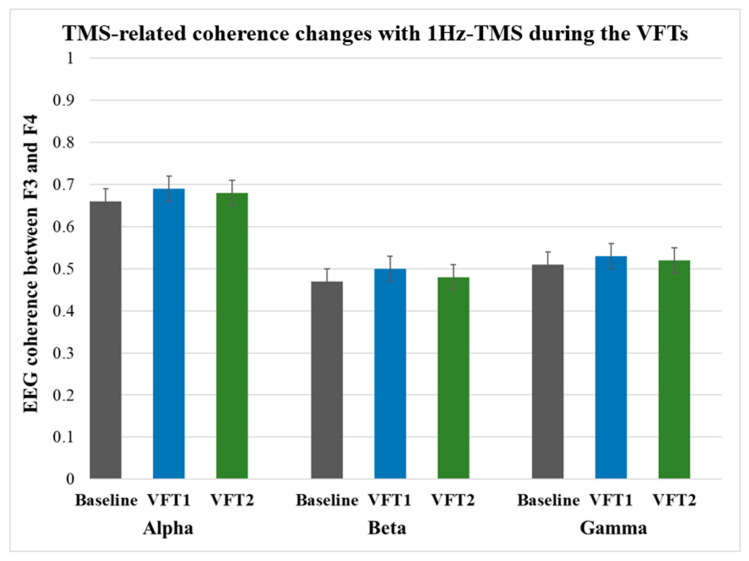
TMS-related coherence changes during the VFTs. The ANOVA showed no significant changes in coherence for all frequency bands during the VFT-1 (alpha: F_1,20_ = 3.16, *p* = 0.101; beta: F_1,20_ = 3.73, *p* = 0.077; gamma: F_1,20_ = 3.23, *p* = 0.098) or VFT-2 (alpha: F_1,20_ = 1.41, *p* = 0.258; beta: F_1,20_ = 1.69, *p* = 0.217; gamma: F_1,20_ = 0.497, *p* = 0.494).

A one-way ANOVA with time showed no significant coherence changes by 1Hz-TMS for the left DLPFC (F3 electrode site) in all frequency bands as follows: coherence changes in alpha band (Baseline vs. VFT-1: F_1,20_ = 3.16, *p* = 0.101; Baseline vs. VFT-2: F_1,20_ = 1.41, *p* = 0.26); beta band (Baseline vs. VFT-1: F_1,20_ = 3.73, *p* = 0.077; Baseline vs. VFT-2: F_1,20_ = 1.69, *p* = 0.22), gamma band (Baseline vs. VFT-1: F_1,20_ = 3.23, *p* = 0.098; Baseline vs. VFT-2: F_1,20_ = 0.50, *p* = 0.49). Furthermore, there were no significant coherence changes between VFT-1 and VFT-2 in each frequency band. Figure 6 shows coherence changes induced by 1Hz-TMS over the left DLPFC during the VFTs for each frequency band.

## 4. Discussion

In the present study, the neurophysiological effects of 1Hz-TMS administered to the right DLPFC were investigated in healthy participants. First, the recruitment curve of N100 component of TEPs by 1Hz-right DLPFC stimulation showed sigmoidal nonlinear changes. Second, 1Hz-TMS to the right DLPFC in the resting-state transiently and significantly increased the TEPs in the alpha, beta, and gamma bands at the stimulation site. Third, 1Hz-TMS to the left DLPFC during VFT was found to increase the TMS-evoked EEG powers transiently and significantly in the alpha and beta bands at the stimulation site. Fourth, 1Hz-TMS to the right DLPFC in the resting-state was found to increase the alpha and beta band coherences transiently and significantly between the right and left DLPFC with TMS stimulation. Fifth, 1Hz-TMS to the left DLPFC during VFTs did not show any specific coherence changes between the left and right DLPFC with TMS stimulation.

### 4.1. Recruitment Curve of TEPs

The recruitment curve obtained in the present experiment changed nonlinearly with stimulus intensity, and the change plateaued at 80% MSO. This result is a positive finding suggesting that the responsivity in TEP detects neurobiological responses rather than artificial changes caused by TMS stimulation.

### 4.2. TMS-Related Power Changes

In previous studies, Valiulis et al. reported that the right prefrontal 1Hz-rTMS induced significant increases of EEG power in alpha, theta, and beta bands in the patients with depression [38], while Noda et al. demonstrated that the left prefrontal 20Hz-rTMS induced a significant increase of EEG gamma power in patients with depression at the stimulation site, which was also associated with an improvement in depressive symptoms [39]. Thus, it may be possible that increases of EEG power in alpha, beta, and gamma bands might reflect the therapeutic mechanism of rTMS for depression regardless of the left or right side of the DLPFC stimulation.

On the other hand, previous studies that examined EEG changes induced by verbal cognitive tasks have shown that alpha power increases in a wide range of brain regions [40], and alpha and beta power increases in the frontal and occipital regions [41]. In the present study, 1Hz-TMS (100 pulses/block) to the left DLPFC during VFTs significantly increased alpha- and beta-band powers, suggesting that TMS stimulation with this protocol does not have a particularly antagonistic interference effect on EEG power changes induced by VFT per se, but may have a synergistic effect. However, in this study, EEG changes due to the VFT itself were not measured as a control condition, and thus further research is needed.

Besides, previous studies that evaluated EEG power changes immediately after high-frequency rTMS to the left M1 of healthy subjects have shown increases in alpha and beta powers in the prefrontal cortex [10,42]. Thus, TMS stimulation of the cortex may induce ubiquitous alpha, beta, and, possibly, gamma-band power changes, independent of the stimulation site. However, Rosanova et al. have also shown that natural frequency responses were observed in the cortex depending on the stimulation site [43], thus further research is needed.

In addition, although the physiological role of gamma-band activity is still not fully understood, previous studies have reported that the generation of gamma-band activity, which is involved in cognitive processes and information processing, is impaired in neuropsychiatric disorders such as schizophrenia and Alzheimer’s disease [44,45,46]. In this context, the present transient increase in gamma power by 1Hz-TMS to the right DLPFC may indicate a physiological mechanism that contributes to cognitive enhancement in healthy subjects and procognitive effects in patients with psychiatric disorders, including depression [47].

### 4.3. TMS-Related Coherence Changes

In the present study, alpha and beta coherences were significantly increased with 1Hz-TMS between both DLPFCs (the F4 and F3 electrode sites) in the resting-state. However, gamma coherence was not significantly changed with 1Hz-TMS. In contrast, there were no significant coherence changes in all frequency bands during the VFTs.

Several studies have examined EEG alpha coherence in healthy subjects and reported that alpha coherence was directly involved in internal information processing and the mechanisms of attention and consciousness [48,49]. Furthermore, these studies have shown an association between the enhancement of these functions and increases in the frontal alpha coherence. These findings suggest that the significant increase in alpha coherence in the prefrontal cortex with 1Hz-rTMS for the right DLPFC may be related to the therapeutic mechanism of depression with TMS therapy.

Previous studies have reported that the frontal intra-hemispheric interdependence in beta band was significantly decreased in patients with depression [50] while the frontal inter-hemispheric coherence in all bands was significantly decreased in patients with depression during emotional face processing [51], compared with healthy controls. Therefore, the present finding that 1Hz-TMS to the right DLPFC significantly increased alpha and beta coherence, albeit transiently, may reflect part of the therapeutic mechanism of 1Hz-rTMS to the right DLPFC for depression. However, since the present study only evaluated the temporary aftereffects of a single session of TMS in healthy subjects, the longer-term neurophysiological effects of 1Hz-rTMS on the right DLPFC in patients with depression should be investigated in the future. 

For gamma coherence, Li et al. reported that the global EEG gamma coherence in patients with depression was significantly higher than that of healthy controls during emotional face processing [52]. However, since no study has reported the gamma coherence differences between patients with depression and healthy controls in resting-state, further studies are awaited.

On the other hand, there was no significant change in coherence in the experiments combining VFT and TMS stimulation. It is possible that the combination of VFT and left prefrontal 1Hz-TMS did not produce significant changes in coherence because the effects of the two stimuli interfered with each other, since VFT or 1Hz-TMS stimulation itself has a distinct effect on increasing frontal coherence [53,54]. Further studies are needed to verify this finding by measuring the EEG in the VFT as a control condition.

### 4.4. Limitations

This study has several limitations. First, since the study subjects in this study were healthy subjects, there was a limitation in examining the treatment mechanism of depression itself. Second, since a sham coil was not used, it was not possible to establish a strict control condition. However, these limitations can be justified, as it is common practice to develop new therapies and explore their therapeutic mechanisms on healthy subjects first in an exploratory way as an open-label pilot study. Third, the analysis of regions of interest in this study was limited to the F3 and F4 electrode sites from the beginning, thus it was not possible to examine TMS-related effects in other areas. Finally, because this study examined aftereffects as acute effects after TMS stimulation, it was not possible to evaluate the long-term effects associated with the entire rTMS treatment for depression. 

### 4.5. Conclusions

The present study demonstrated that low-frequency 1Hz TMS to the right DLPFC in healthy participants resulted in a significant increase in spectral power in the alpha and beta bands.

Likewise, 1Hz-TMS to the right DLPFC induced a significant increase in alpha and beta coherences between the right and left DLPFC in healthy participants. Collectively, these findings suggest parts of the therapeutic mechanisms of 1Hz-rTMS to the right DLPFC for depression. Lastly, the present study warrants future research that investigates the neurophysiological effects of various types of TMS treatment, providing unique and informative perspectives on the pathophysiology underlying neuropsychiatric disorders as represented by major depressive disorders and schizophrenia [55].

## Data Availability

Data sharing not applicable. No new data were created or analyzed in this study. Data sharing is not applicable to this article.
